# A randomized sham‐controlled study of pulmonary vein isolation in symptomatic atrial fibrillation (The SHAM‐PVI study): Study design and rationale

**DOI:** 10.1002/clc.24066

**Published:** 2023-06-13

**Authors:** Rajdip Dulai, Stephen S. Furniss, Neil Sulke, Nick Freemantle, Pier D. Lambiase, David Farwell, Neil T. Srinivasan, Stuart Tan, Nikhil Patel, Adam Graham, Rick A. Veasey

**Affiliations:** ^1^ Cardiology Research Department, Eastbourne District General Hospital East Sussex Hospitals NHS Trust Saint Leonards‐on‐Sea East Sussex UK; ^2^ Institute of Cardiovascular Science University College of London London UK; ^3^ CVS Healthcare Ltd. Eastbourne UK; ^4^ Institute for Clinical Trials and Methodology University College London London UK; ^5^ Mid and South Essex NHS Foundation Trust The Essex Cardiothoracic Centre Essex UK; ^6^ Nottingham University Hospital NHS Trust Nottingham UK

**Keywords:** ablation, atrial fibrillation, catheter ablation, cryoablation, placebo, pulmonary vein isolation, sham

## Abstract

**Introduction:**

Pulmonary vein (PV) isolation has been shown to reduce atrial fibrillation (AF) burden and symptoms in patients. However, to date previous studies have been unblinded raising the possibility of a placebo effect to account for differences in outcomes.

**Hypothesis & Methods:**

The objective of this study is to compare PV isolation to a sham procedure in patients with symptomatic AF. The SHAM‐PVI study is a double blind randomized controlled clinical trial. 140 patients with symptomatic paroxysmal or persistent AF will be randomized to either PV isolation (with cryoballoon ablation) or a sham procedure (with phrenic nerve pacing). All patients will receive an implantable loop recorder. The primary outcome is total AF burden at 6 months postrandomisation (excluding the 3 month blanking period). Key secondary outcomes include (1) time to symptomatic and asymptomatic atrial tachyarrhythmia (2) total atrial tachyarrhythmia episodes and (3) patient reported outcome measures.

**Results:**

Enrollment was initiated in January 2020. Through April 2023 119 patients have been recruited. Results are expected to be disseminated in 2024.

**Conclusion:**

This study compares PV isolation using cryoablation to a sham procedure. The study will estimate the effect of PV isolation on AF burden.

## Graphical abstract

The SHAM‐PVI study timeline. One hundred forty patients will be enrolled and randomized in a 1:1 ratio to receive cryoablation or a sham procedure. All patients will receive an implantable loop recorder. Outcomes will be assessed at 3 and 6 months post‐randomisation with the first 3 months constituting the blanking period.

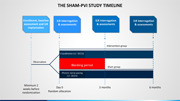

## 1 INTRODUCTION

Atrial fibrillation (AF) is the most common cardiac arrhythmia with an 8.5% prevalence in men and 7.1% prevalence in women over the age of 55.^1^ It is estimated there are 8.8 million adults with AF in the European Union.^1^

Catheter ablation has been shown to reduce the occurrence of AF and improve quality of life and symptoms in patients with symptomatic AF when compared to medical therapy, for example the CABANA study showed significant improvements in quality of life and symptoms.^2^, ^3^ However previous trials involving catheter ablation have not been blinded raising the possibility of a placebo effect accounting for the differences in outcomes.

The ORBITA trial (Percutaneous intervention vs placebo in angina patients) and SYMPLICITY HTN‐3 (renal denervation vs sham procedure in resistant hypertension) trials have shown that placebo controlled trials for device therapy are safe and feasible. Indeed both trials showed a placebo effect in device therapy, which had not been accounted for previously.^4^, ^5^

This study is an investigator led randomized double blind trial comparing catheter ablation to sham therapy in patients with symptomatic persistent or paroxysmal AF.

## 2 METHODS

The objective of this study is to evaluate the effectiveness of pulmonary vein (PV) isolation vs a sham procedure in patients with symptomatic persistent or paroxysmal AF. The null hypothesis predicts that PV isolation has no effect on AF burden compared to a sham procedure.

Paroxysmal AF (PAF), also termed intermittent AF, is defined as any episode of AF that terminates spontaneously or with intervention in less than 7 days.^6^ Persistent AF is defined as any continuous AF episode that is sustained beyond 7 days.^6^ Long‐term persistent AF is defined as any continuous AF episode lasting more than 12 months in duration and patients in permanent AF are defined as such when the clinician and patient make a joint decision to stop further attempts at maintaining sinus rhythm.^6^

The major inclusion criteria for the study comprise patients with symptomatic paroxysmal or persistent AF despite at least one antiarrhythmic drug (AAD Type I or III, including β‐blocker and AAD intolerance). The major exclusion criteria include long‐term persistent AF (any continuous AF episode lasting more than 1 year), prior left atrium (LA) catheter or surgical AF ablation, patients with other arrhythmias requiring ablative therapy, LA ≥ 5.5 cm and ejection fraction (LVEF) less than 35% (Table 1). Any patient enrolled in the study who withdraws their consent will be removed from the study, but at enrollment their consent will be sought to use the data already recorded. For participants who are lost to or do not attend follow‐up, data shall be obtained from hospital medical records and/or primary care records where possible.

**Table 1:** Inclusion and exclusion criteria.

Inclusion criteria
Age greater than or equal to 18 years
Symptomatic paroxysmal or persistent atrial fibrillation despite at least one antiarrhythmic drug (AAD Type I or III, including β‐blocker and AAD intolerance)
Referred for catheter ablation
Exclusion criteria
Long term persistent AF (any continuous AF episode lasting more than 1 year)
Prior left atrium catheter or surgical atrial fibrillation ablation
Patients with other arrhythmias requiring ablative therapy
Left atrium (LA) ≥ 5.5 cm
Left ventricular ejection fraction (LVEF) less than 35%
Any cardiac surgery or percutaneous coronary intervention (PCI) within 3 months before enrollment.
Awaiting cardiac surgery or PCI
Myocardial infarction within 3 months before enrollment
Stroke or transient ischemic attack (TIA) within 3 months before enrollment
Unstable angina
Any significant congenital heart defect corrected or not (including atrial septal defects or PV abnormalities) but not including patent foramen ovale
Any condition contraindicating chronic anticoagulation
Any untreated or uncontrolled hyperthyroidism or hypothyroidism
Severe chronic kidney disease (stage V, requiring or almost requiring dialysis, glomerular filtration rate (GFR) < 15 ml/min)
Patients with prosthetic valves
Pregnant or breastfeeding women
Medical conditions limiting expected survival to <1 year
History of claustrophobia or panic attacks

Outcome measures will be evaluated at 3 months postrandomisation and 6 months postrandomisation (Table 2). The first 3 months postrandomisation will constitute the blanking period. The primary outcome measured is the comparison of AF burden using continuous monitoring at 6 months post‐randomisation between the intervention group and control group. Key secondary outcomes include time to atrial tachyarrhythmia and patient reported outcome measures (Table 3).

**Table 2:** Schedule of interventions and assessments.

	Enrollment	Day 0/Procedure day	3 Months	6 Months
Consent	X			
AF symptom review	X		X	X
Clinical examination	X		X	X
Medication review	X		X	X
Adverse event review		X	X	X
Echo (if not performed in last 12 months)	X			
ECG	X	X	X	X
Questionnaires		X	X	X
ILR implantation (if not already implanted)	X			
ILR interrogation		X	X	X
Procedure (ablation/sham)		X		
Blinding assessment		X	X	X

Abbreviation: ILR, implantable loop recorder.

**Table 3:** Secondary outcomes.

Time to any atrial tachyarrhythmia stratified by length of episode (more than 30 s/more than 7 days)
Time to symptomatic atrial tachyarrhythmia
Number of atrial tachyarrhythmia episodes (symptomatic and asymptomatic) in the follow‐up period in each group stratified by length of episode (more than 30 s/more than 7 days)
Comparison of medical treatment in each group in the follow‐up period
Comparison of health related quality of life in each group (SF‐36)
Comparison of AF specific quality of life score between each group; Atrial Fibrillation Effect on Quality‐of‐Life (AFEQT) Questionnaire, Mayo AF Symptom Inventory (MAFSI) and EHRA class
Comparison of unscheduled use of health care services during follow‐up
Procedure related complications/adverse events between each group

## 3 METHODS

**Figure 1:**
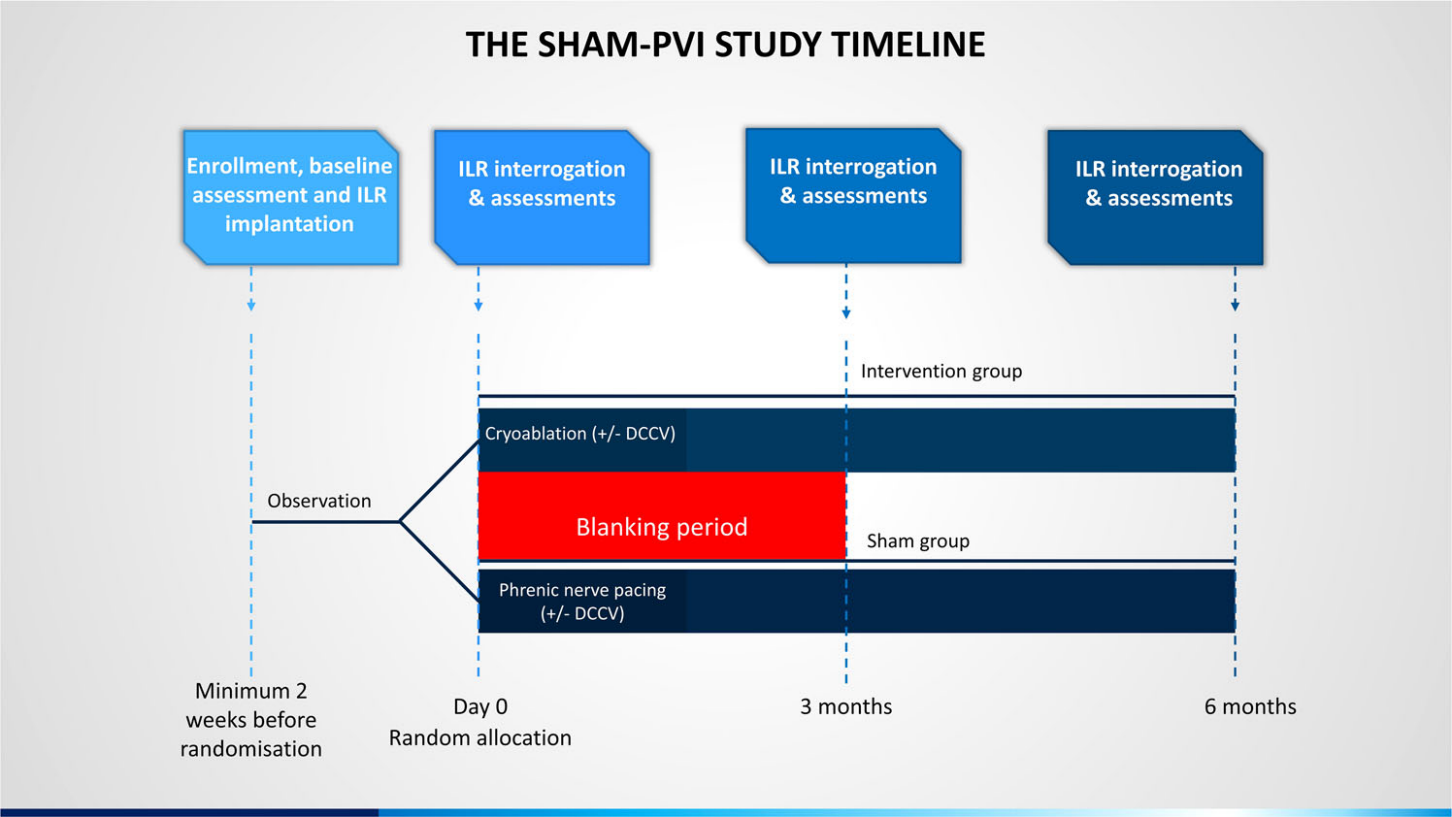
The SHAM PVI study timeline. One hundred fourty patients will be enrolled and randomized in a 1:1 ratio to receive cryoablation or a sham procedure. All patients will receive an ILR. Outcomes will be assessed at 3 and 6 months post‐randomisation with the first 3 months constituting the blanking period. DCCV, Direct current cardioversion; ILR, implantable loop recorder.

This is a double blind, randomized sham controlled study commenced in December 2019 (Figure 1). The committee members are listed in the Supporting Information Material (Supporting Information: Appendix 1). Patients meeting the inclusion and exclusion criteria are recruited from hospitals within East Sussex Healthcare NHS Trust and Mid and South Essex NHS Foundation Trust (Supporting Information: Appendix 2). 140 patients meeting the inclusion and exclusion criteria will be recruited. Ethical approval has been obtained from the West Midlands—South Birmingham Committee (19/WM/0361).

Following recruitment and baseline measurements, all patients will undergo an implantable loop recorder (ILR) implantation (Reveal LINQ, Medtronic Inc.) if this has not been inserted previously. It is recommended the ILR be inserted at least 2 weeks before the main procedure day or on the main procedure day dependent on covid‐19 restrictions.

### 3.1 Implantable loop recorder insertion

A Medtronic Reveal LINQ loop recorder will be inserted as per manufacturer guidelines at study enrollment. The device settings will be optimized to record all AF episodes longer than 2 min (Supporting Information: Table 1). The device is able to wirelessly transmit all ECG recordings and activated episodes on a daily basis. The AF algorithm has a reported sensitivity of 97.4% and positive predictive value of 73% however all recordings will be manually reviewed by a 3‐person adjudication committee blinded to patient group.^7^, ^8^

### 3.2 Pre‐procedure medication management

AADs will be discontinued five half‐lives (up to 5 days) before the procedure, except for Amiodarone, which will be discontinued 8 weeks before procedure day. Procedures will be performed on uninterrupted anticoagulation. Patients will remain on anticoagulation for the duration of the study.

### 3.3 Randomization

Participants will be randomly assigned in a 1:1 ratio to undergo either catheter ablation ± DCCV (if in AF) or a sham procedure ± DCCV (if in AF). A computerized central blocked randomization design will be generated and stratified according to type of AF (paroxysmal/persistent). Randomization blocks will be performed with “ralloc,” Stata's randomization process v.16.0. The block sizes will not be disclosed to study investigators, to ensure concealment.

The randomization sequence and study‐group assignments will be prepared and placed in sequential numbered sealed, opaque envelopes by a fellow with no involvement in the execution of the trial. The envelopes will be kept securely by a sponsor administrator not involved in the conduct of the study.

The allocation will remain concealed until after sedation has been achieved at the time of the procedure.

### 3.4 Sedation and blinding

During each procedure patients will be given over‐the‐ear headphones playing music to prevent hearing of communication between cath‐lab staff. Patients will then be sedated during the procedure using opiates and benzodiazepines and have eye coverings if necessary. After the procedure, all nursing staff, physicians and other health care professionals performing the procedure will have no further contact with the patient during follow‐up. Health care professionals or research staff involved in the patient care post‐procedure and during follow‐up will be blinded to the treatment strategy (Supporting Information: Appendix 3 and Supporting Information: Figure 1).

Participant blinding will be assessed at the time of discharge, 3 months and at 6 months follow‐up. Participants will be asked to guess one of the following: (1) ablation, (2) placebo, (3) Don't know. Participants will also be asked to state the certainty of their answers on a grade scale of 1−5 with 5 being most sure.

Staff members will also be asked at discharge, 3 months and at 6 months follow‐up to guess the patient treatment allocation.

### 3.5 Cryoablation procedure

At the beginning of the procedure x2 femoral venous access will be achieved using ultrasound guidance. If the patient is in AF then DCCV will be undertaken to cardiovert to sinus rhythm.

A multipolar catheter will then be placed in the coronary sinus. The LA will then accessed via trans‐septal puncture or patent foramen ovale. Following left atrial access, IV heparin will be administered as sequential boluses maintaining activated clotting time more than 300 s. Thereafter the TS sheath will be exchanged with a steerable 15 Fr sheath (Flexcath, Medtronic). A 28 mm cryoballoon catheter (Arctic Front Advance, Medtronic) will be advanced through the steerable sheath into the LA with a guide wire or the Achieve mapping catheter in the central lumen.

The cryoballoon will be positioned in the ostium of each PV using fluoroscopic guidance and contrast injection with minimal or no dye leak on injection after inflation.

Before ablation of the right PVs, the multipolar catheter will be placed in the right subclavian vein to pace the right phrenic nerve (10–20 mA at 1.0–2.0 ms pulse width at a cycle length of 1000 ms). Ablation will be immediately terminated upon any perceived reduction in the strength of diaphragmatic contraction.

Cryoablation in each PV will be applied for a minimum duration of 180 s and maximum duration of 240 s. If the temperature has not reached −40 degrees by 60 s then this will be deemed to be an ineffectual ablation and the ablation will be stopped and the balloon repositioned. A further 2 attempts at ablation will be allowed. Entrance and exit block will also be confirmed and if the operator fails to isolate the PV (excluding common ostia) after a minimum of 3 attempted cryoballoon applications then focal ablation with the 8 mm cryocatheter (Freezor Max) targeted to sites of LA‐PV breakthrough will be permitted at operator discretion.

At the end of the procedure once sheaths have been removed all patients will have a three‐way stopcock suture to achieve haemostasis.

### 3.6 Sham procedure

After x2 venous access has been achieved using ultrasound guidance, DCCV will be undertaken if the patient is in AF. A 5‐Fr pacing catheter will then be placed at the right subclavian vein to pace the phrenic nerve (10–20 mA at 1.0–2.0 ms pulse width at a cycle length of 1000 ms). The phrenic nerves will be paced for 4 min on four occasions during the procedure. Operators are advised to keep the patient in the catheter lab for a minimum of 1 h.

At the end of the procedure once sheaths have been removed all patients will have a three‐way stopcock suture to achieve haemostasis.

### 3.7 Follow‐up

AF episodes will be managed medically as per ESC guidelines during the follow‐up phase.^9^ Only 1 DCCV will be permitted in each participant during the follow‐up phase. Antiarrhythmic medications may be restarted depending on the reoccurrence of AF and symptoms. Antiarrhythmic medications will be stopped 5 half‐lives before follow‐up at 3 months. The use of Amiodarone is discouraged. If patients have an alternative indication for beta blocker medications (e.g., hypertension or heart failure) then this may be continued. Patients will undergo scheduled follow‐up at 3 and 6 months (Table 2) after randomization to record

- AF symptom status
- Medication review
- Adverse event review
- ECG
- Implantable loop recorder interrogation
- Blinding assessment.

### 3.8 Sample size calculation

The study will be powered to address the primary hypothesis that PV isolation will reduce the total AF burden compared to patients undergoing a sham procedure at 6 months post‐randomisation. The CASTLE AF trial reported AF burden using continuous monitoring at 3 months in the ablation group to be 27% and 51% in the pharmacological group. At 6 months the AF burden in the ablation group was reported to be 23% versus 51% in the pharmacological group.^10^ Based on previous published data and the clinical investigators experience we estimated the AF burden in the intervention group to be 25% at 6 months follow‐up and in the control group to be 50%. We assume a standard deviation of 48%. Based on these data and assumptions with 80% power and two‐sided 0.05 *α* 118 patients will be required in total to be recruited. We will recruit 140 patients (70 in each group) to take in to account withdrawals and patients lost to follow‐up.

### 3.9 Planned analysis

The full analysis schedule will be described before database lock in a statistical analysis plan. Briefly, the primary analysis will compare the AF burden between each group at 6 months follow‐up post‐randomisation using a generalized mixed model, including baseline and post intervention observations for each subject and parameterized to identify the period (baseline or postrandomisation) and the randomized condition in the post treatment period. Observations within a patient will be linked with a random intercept term and the denominator degrees of freedom for the principal analysis will be derived from the number of patients rather than the number of observations. *p* < .05 will be considered statistically significant. We will utilize a treatment policy estimand.

### 3.10 Screening and recruitment

A total of 140 participants will be recruited into this trial. Those screened but not recruited will not be disadvantaged in their usual care. An anonymised record of those patients screened, as well as their reasons for not participating in the trial (but no other information) will be kept in the screening log.

### 3.11 Data monitoring and safety committee (DMSC)

An independent DMSC will be convened containing 3 members which will meet to provide independent advice on study conduct and safety issues (Supporting Information: Appendix 4). Meetings will be held approximately annually or as required throughout the duration of the trial. Safety data will be studied after 70 patients have received treatment and completed the study, or 1 year after the first patient is randomized, whichever occurs sooner. Meetings will also be held as necessary should any urgent issues occur.

## 4 RESULTS

Through to April 2023 119 patients have been enrolled. Preliminary baseline characteristics on 105 of the 119 patients enrolled through April 2023 is presented in Table 4. Full recruitment is expected to be completed in September 2023 with dissemination of results in 2024.

**Table 4:** Preliminary baseline characteristics on 105 of the 119 patients enrolled through April 2023.

Characteristic	Randomized patients (*N* = 105)
Age	66.4 ± 8.7
Male sex**—** *N* (%)	76 (72.4)
Body mass index	28.9 ± 4.0
Blood pressure (mm Hg)	
Systolic blood pressre	135 ± 17.5
Diastolic blood pressure	81.7 ± 13.5
Mean time since 1st diagnosis of AF (months)	44.5 ± 51.7
Type of atrial fibrillation *N* (%)	
Paroxysmal atrial fibrillation	21 (20.0)
Persistent atrial fibrilaltion	84 (80.0)
Previous cardioversion *N* (%)	68 (64.8)
Mean number of cardioversions	1.8 ± 1.6
Previous hospitalization for AF *N* (%)	41 (39.0)
Left atrial diameter (mm)	41.5 ± 5.0
Left ventricular ejection fraction (%)	54.3 ± 5.3
CHA2DS2‐VASc score	2.0 ± 1.5
Co‐morbidities *N* (%)	
Coronary artery disease	27 (25.7)
Myocardial infarction	9 (8.6)
COPD/Asthma	8 (7.6)
Type 2 diabetes	11 (10.5)
Thyroid disease	3 (2.9)
Hypertension	52 (49.5)
CVA/TIA	2 (1.9)
Heart failure	11 (10.5)
*New York Heart Association Class*	
1	99 (94.3)
2	6 (5.7)
AF medications *N* (%)	
Beta blocker	97 (92.4)
Calcium channel blocker	7 (6.7)
Digoxin	8 (7.6)
Flecainide	23 (21.9)
Sotalol	21 (20)
Amiodarone	25 (23.8)
Dronedarone	9 (8.6)
Propafenone	2 (1.9)
Smoking history	
Never	52 (49.5)
Current	4 (3.8)
Ex‐smoker	49 (46.7)
Average alcohol intake per week (units)	7.0 ± 9.2
Preprocedure ILR monitoring (days)	43.0 ± 79.2

Abbreviation: ILR, implantable loop recorder.

## 5 DISCUSSION

To date there has not been a double‐blind randomized controlled trial comparing PV isolation to a sham procedure. Given this, physicians have advocated for a sham‐controlled study in patients with AF to fully evaluate the efficacy of PV isolation to account for any placebo effect.^11^, ^12^, ^13^ The study is one of two ongoing full scale clinical trials examining the placebo effect of PV isolation.^14^, ^15^

In this study, healthcare professionals and physicians post‐procedure will be blinded to treatment strategy. Although previous studies examining catheter ablation for PV isolation have included end point blinded adjudication committees, the lack of blinding of physicians treating patients may confound results. As physicians post‐procedure will be blinded to the treatment received in this study, all patients will be treated equally on the same pathway, thus minimizing bias.

The first 3 months post‐procedure constitute the blanking period as recommended by the 2017 HRS/EHRA/ECAS/APHRS/SOLAECE expert consensus statement on catheter and surgical ablation of AF.^15^ Although outcomes are measured at 3 months, arrhythmia‐based outcomes and burden in the blanking period will be censored.

The study is powered to address the primary hypothesis that AF ablation results in a significant AF burden reduction compared to a sham procedure. Quality of life and AF symptoms will also be assessed as secondary outcomes. The sample size calculation and standard deviations are based on CASTLE‐AF data and the clinical investigators own experience, specifically continuous monitoring data from The Eastbourne District General Hospital AF Ablation Registry.^10^ At present, no sub‐studies are planned. An independent DMSC has been convened to primarily monitor study conduct and safety issues. There are no stopping rules for overwhelming superiority.

The study is limited to 6 months follow‐up. Previous studies examining PV isolation with a longer follow‐up have had high crossover rates, which affects the interpretation of results for example, In the CABANA trial 9% of patients in the ablation group did not undergo ablation and 22.3% of the patients in the medical therapy group underwent ablation.^2^ Given the shorter duration of follow‐up in this study, crossovers are not expected however the sample size has been increased to 140 to take in‐to account a potentially high number of withdrawals. Additionally, as follow‐up is limited to 6 months, the use of amiodarone is discouraged given its extremely long plasma half‐life.^16^

A challenge of the study to date has been the recruitment of patients during the COVID‐19 pandemic which has delayed study recruitment. However, to limit any potential effect of the COVID‐19 pandemic, recruitment was paused in March 2020 and restarted in July 2021. Protocol changes were made to facilitate follow‐up during the COVID‐19 pandemic. The ILR insertion timing recommendation was reduced to a minimum of 2 weeks before the ablation/sham procedure date. During 2021, there was uncertainty regarding further surges in COVID‐19 and the impact this would have on the study. Thus the ILR insertion was also allowed to be performed at the time of the ablation/sham procedure. This would have reduced the requirement for patients to isolate before entering hospital sites. However to date, this has not been required and all patients have had their ILR inserted before the ablation/sham procedure allowing a pre‐procedure AF burden in all patients.

### 5.1 Limitations

One of the limitations of this study is that follow‐up is only 6 months. This is shorter than previous clinical trials of AF ablation, which have a minimum follow‐up of at least 1 year, although all previous studies have been unblinded.^3^, ^17^, ^18^ A 6 month follow‐up was selected as this is the shortest period of time required to see the treatment effect of PV isolation. In addition, we considered patient feedback when designing the study. The majority of patients reported that they would unlikely consent for a study involving a sham procedure that lasted 1 year as opposed to 6 months. Extending follow‐up to 1 year may also cause a selection bias as patients who are mildly symptomatic or have very infrequent episodes of AF would be the patients who accept being in the study as opposed to patients who are more symptomatic.

Another limitation is that this study only uses cryoablation and ablation is limited to PV isolation only. This is unlikely to affect the results given that additional ablation including complex fractionated electrogram and linear ablation has not been shown to be superior to PV isolation alone in large randomized controlled trials.^19^

## 6 CONCLUSION

The SHAM‐PVI study is a double‐blind randomized controlled study comparing PV isolation and a sham procedure. The study will evaluate AF burden and patient‐reported outcomes. The study will provide evidence on the placebo effect, if any, of PV isolation.

## CONFLICT OF INTEREST STATEMENT

N. S. is a trustee of Eastbourne Cardiology Research Charity Fund. PDL receives research grants from Medtronic, Abbott, and Boston Scientific. N. F. receives consulting fees from ALK, Sanofi Aventis, Gedeon Richter, Abbott, Galderma, AstraZeneka, Ipsen, Vertex, Thea, Novo Nordisk, Aimmune and sits on a DSMB for Orion. NTS receives research grants from Abbott. R. A. V. receives research grants from Medtronic. The remaining authors declare no conflict of interest.

## Supporting information

Supporting information.

## Data Availability

The data that support the findings of this study are available from the corresponding author upon reasonable request.
