# Supplementary material for: A randomized sham‐controlled study of pulmonary vein isolation in symptomatic atrial fibrillation (The SHAM‐PVI study): Study design and rationale
Source: Clin Cardiol. 2023 Jun 13;46(8):973–80. doi: 10.1002/clc.24066 (PMC10436780; doi:10.1002/clc.24066)
Supplement: Supplementary file 1 — Supporting information. [file CLC-46-973-s001.docx]

**Supplementary Appendix**

Supplementary Appendix 1 – committee groups

Steering committee: Dr Rajdip Dulai, Dr Stephen Furniss, Dr Rick Veasey, Dr Thomas Keeble (independent chair)

Statistician: Prof Nicholas Freemantle

Blinded adjudication committee: Dr Rajdip Dulai, Dr Stephen Furniss, Dr Neil Sulke, Dr Adam Graham (from July 2022)

Independent data monitoring & safety committee: Dr Senthil Kirubakaran, Dr Steven Podd, Dr Conn Sugihara

Supplementary Appendix 2 – Study sites

1)East Sussex Healthcare NHS Trust (Principle investigator Dr Rick Veasey)

- Eastbourne District General Hospital (ablation / sham procedure site)
- Conquest hospital

2)Mid and South Essex NHS Foundation Trust (Principle investigator Dr David Farwell)

- Essex Cardiothoracic Centre (ablation / sham procedure site)
- Basildon University Hospital
- Southend University Hospital
- Broomfield Hospital

**Supplementary Appendix 3 – further information on blinding**

No procedural details will be inputted into the patients electronic care records or written notes. Procedure details will be kept securely in a locked cabinet by an administrator not involved in the study and will only be available in the case of an emergency and a need to deblind the study team or patient. Post procedure all patients will undergo the same recovery checks and discharge instructions as per the local hospital policy for ablation procedures. All patients will receive the same discharge letter (Supplemenatry Figure 1).

**Supplementary Appendix 4 - Defined adverse events**

The following procedural adverse events are defined:

**Expected adverse events**

- Bruising, haematoma, vascular injury not requiring intervention
- Pericardial or pleural effusion not requiring intervention
- Headache
- Infection (e.g. pneumonia)
- Pulmonary oedema
- Temporary phrenic nerve damage
- Pericarditis

**Expected serious adverse events**

- Pericardial effusion requiring intervention
- Vascular complication requiring blood transfusion or intervention
- Phrenic nerve palsy (permanent)
- Shortness of breath more than 1 week from the procedure
- Chest pain more than 1 week from the procedure
- Pulmonary vein stenosis
- Death
- Oesophageal atrial fistula
- Stroke or TIA

**Supplementary Figure 1:** Example discharge summary given to all patients


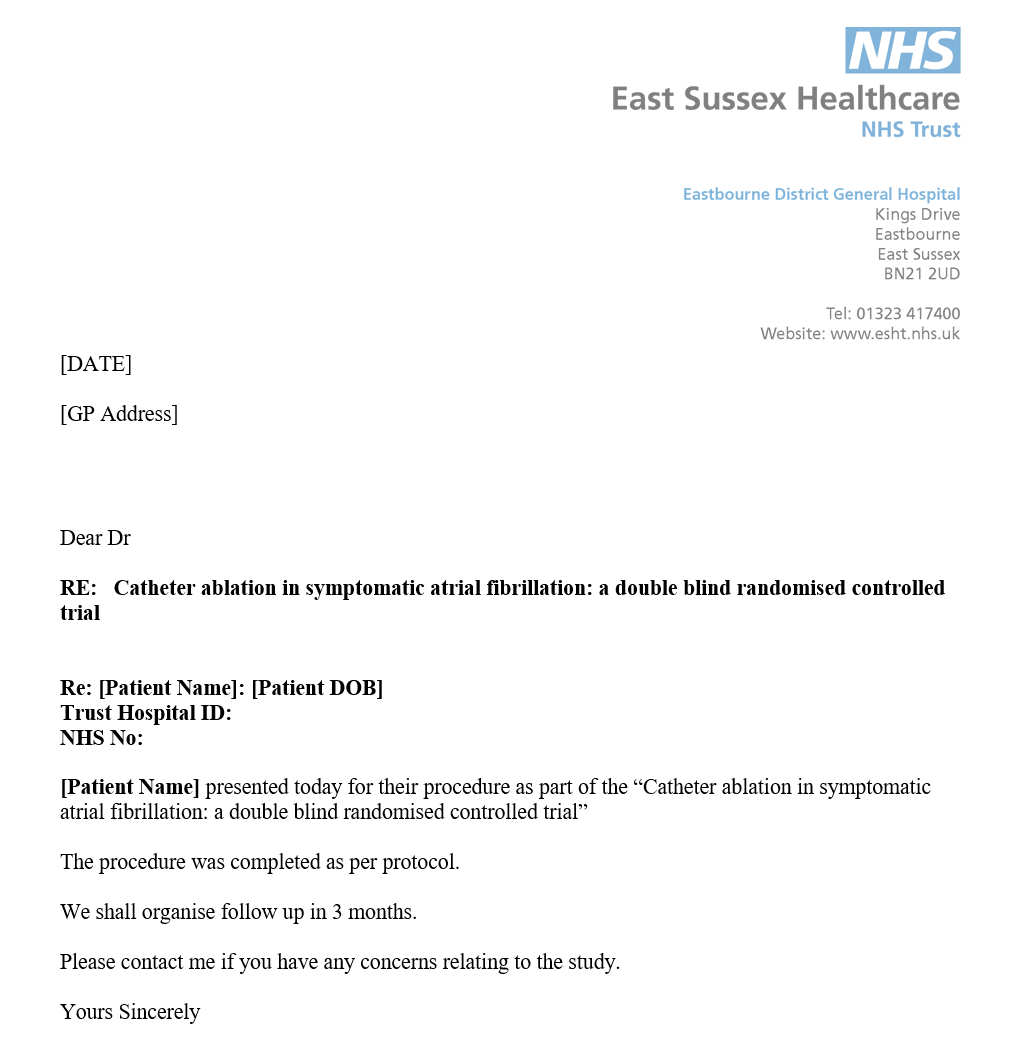


**Supplementary Table 1:** Implantable loop recorder programming settings

| **Parameter** | **Setting** |
| --- | --- |
| AF detection threshold | Balanced Sensitivity |
| Ectopy rejection | Nominal |
| Episode storage threshold | All |
| Tachycardia detection | Interval = 400 ms duration 16 beats |
